# Anti-Chol-1 Antigen, GQ1bα, Antibodies Are Associated with Alzheimer’s Disease

**DOI:** 10.1371/journal.pone.0063326

**Published:** 2013-05-23

**Authors:** Toshio Ariga, Masaru Kubota, Makoto Nakane, Kenji Oguro, Robert K. Yu, Susumu Ando

**Affiliations:** 1 Institute of Molecular Medicine and Genetics, Medical College of Georgia, Georgia Regents University and VA Medical Center, Augusta, Georgia, United States of America; 2 Oyama Orthopedics and Internal Medicine Hospital, Oyama, Tochigi, Japan; 3 Department of Neurosurgery, Teikyo University School of Medicine, University Hospital Mizonokuchi, Kawasaki, Kanagawa, Japan; 4 Tokyo Metropolitan Geriatric Hospital and Institute of Gerontology, Itabashi-ku, Tokyo, Japan; Oregon Health & Science University, United States of America

## Abstract

The interaction of amyloid β-proteins (Aβ) with membrane gangliosides has been reported to be an early event in Aβ fibril formation in Alzheimer’s disease (AD). Neuronal degeneration in AD has been postulated to be associated with the presence of anti-ganglioside antibodies in patient sera. Using an enzyme-linked immunosorbent assay (ELISA) and high-performance thin-layer chromatography (HPTLC) immunostaining, sera from 27 individuals (10 with AD, 6 with vascular dementia (VD), and 11 non-demented age-matched pathological controls) were examined in order to detect anti-glycosphingolipid (GSL) antibodies, including anti-cholinergic-specific antigen (Chol-1α; GQ1bα) antibodies. All sera had natural antibodies against ganglio-N-tetraosyl gangliosides (brain-type gangliosides). However, sera of demented patients with AD and VD had significantly higher titers of anti-GSL antibodies than those in age-matched pathological controls. Although most serum antibodies, including anti- GM1, -GT1b, -GQ1b, -GQ1bα, were of the IgM type, the presence of the IgG type antibodies was also significantly elevated in the sera of demented patients with AD. Anti-GT1b antibodies of the IgG type were elevated in AD (90%, 9 of 10 cases) and VD (100%), respectively. Most surprisingly, anti-GQ1bα antibodies (IgM) were found in 90% (9/10) and 100% (6/6) in the sera of patients with AD and VD, respectively. Since GQ1bα is present in the cerebral cortex and hippocampus, the presence of anti-GQ1bα antibodies may play an important role in disrupting cholinergic synaptic transmission and may participate in the pathogenesis of dementia. We conclude that elevated anti-GSL antibody titers may be useful as an aid for clinical diagnosis of those dementias.

## Introduction

Alzheimer's disease (AD) is the most common type of dementia with clinical symptoms that include abnormalities with memory, judgment, and thinking. The disease interferes with the activities of daily living and becomes progressively worse; eventually the patients may fail to recognize family members, wander away, and need total care. Although histological changes occur to some extent in brains during normal aging, the changes in AD brains are more prominent. The disease also affects more women than men [1], [2], [3]. Vascular dementia (VD) or multi-infarct dementia is a degenerative cerebrovascular disease and refers to dementia associated with problems in cerebral blood flow. It occurs when the blood supply carrying oxygen and nutrients such as glucose to the brain is interrupted by an occlusion of the cerebrovascular system. VD generally affects people between the ages between 60 and 75 years, and affects more men than women. Like AD, VD also affects memory, thinking, language, judgment, and behavior. These symptoms may be tempered with appropriate medications, but there is no cure for these forms of dementia.

Gangliosides are known to play an important role in neural development and regeneration [4]. Anti-ganglioside antibodies have been described in the sera of patients with peripheral neuropathy and many other neurological diseases [5], [6], and have been shown to be responsible, in whole or in part, for the pathogenic mechanisms of these disorders. For example, passive transfer of anti-GD1a antibody severely inhibited axon regeneration after peripheral nervous system (PNS) injury in mice [7]. Chapman et al. [8] examined the presence of serum antibodies in patients with AD and was the first to report that the presence of anti-ganglioside antibodies was associated with this disease. A significantly elevated level of antibodies specific to GM1 was found in the sera of patients with AD compared with age-matched normal controls. In addition, a high level of serum antibodies to GM1 was found in patients with multi-infarct dementia and Parkinson's disease (PD) with dementia, but not in non-demented patients with other neurodegenerative diseases, suggesting the involvement of anti-GM1 antibodies in dementia associated with these diseases. Those findings may reflect a specific change in ganglioside metabolism that is associated with neurodegenerative processes underlying AD and other causes of dementia [2], [8]. In the present study, we examined anti-glycosphingolipid (GSL) antibodies in the sera of patients with AD and VD compared to age-matched controls. We found that patient sera have IgM type anti-brain ganglioside antibodies and anti- GQ1bα, a specific Chol-1 antigen, antibodies. These findings may have diagnostic value for demented patients with AD and VD, and contribute to an understanding of the pathogenic mechanisms of these disorders.

## Methods

### Ethics Statement

The study was approved by the Ethics Committee of the Oyama Orthopedics and Internal Medicine Hospital. Participants gave written informed consent.

### GSL Antigens and Patient Sera

**Figure 1:**
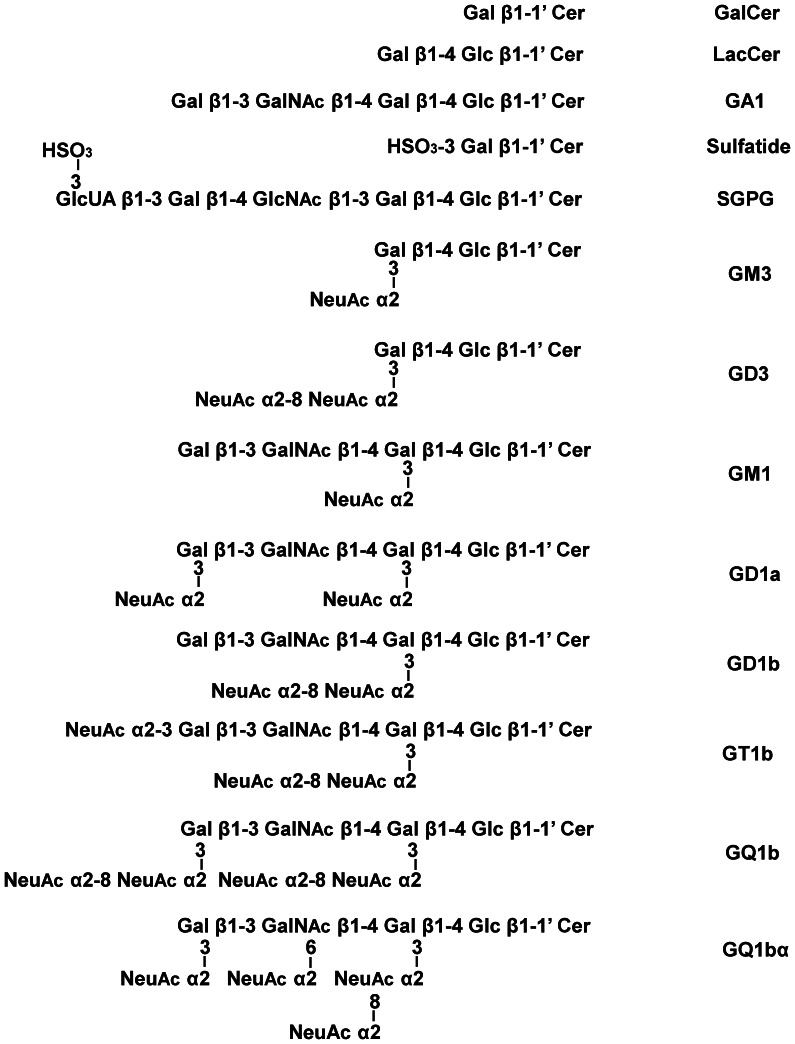
Structures of glycosphingolipids cited in this study. Symbols used for gangliosides and other glycosphingolipids are based on the system of Svennerholm and the nomenclature recommended by IUPAC.

Chol-1α- specific antigen, GQ1bα, was isolated from bovine brains [9], [10]. Other gangliosides, sulfatide, and neutral GSLs were isolated in our laboratories using established procedures [11], [12], [13], [14]. Fig. 1 shows the structures of GSLs cited in the present study. The sera from 27 individuals, 10 with AD, 6 with VD, and 11 with age-matched controls were obtained at the above hospital. The severity of cognitive impairment was evaluated using the Mini-Mental State Examination (MMSE). Magnetic resonance imaging (MRI) was performed on all patients. Diagnosis of AD and VD was based on criteria of the Diagnostic and Statistical Manual of Mental Disorders (DSM-IV). All patients with AD and VD demonstrated hippocampal or cortical involvements with MRI (data not shown).

### Enzyme-linked Immunosorbance Assay (ELISA)

ELISA was carried out according to the method of Kohriyama et al. [15]. Twenty-five microliters of a methanolic solution of a GSL (1 µg/mL), equivalent to 25 ng, was added to each well of a 96-well, flat-bottom polystyrene microtiter plate (Immulon 1B, Thermo, Milford, MA), and the solution was dried at room temperature overnight. One hundred microliters of 1% bovine serum albumin (BSA)/phosphate buffered saline (0.5 M PBS; pH 7.4) were added to each well and the sample was incubated at 37°C for 30 min. After decanting and washing each well with PBS five times, 100 µL of the test serum (diluted 1∶400 with 1% BSA/PBS) were added and incubated for 2 hrs. After washing as described above, 100 µL of peroxidase-conjugated anti-human IgG or IgM antibodies (Cappel, West Chester PA; diluted 1∶3,000 with 1% BSA/PBS) were added and the sample incubated for 2 hrs and washed. The enzyme activities were measured by adding 100 µL of o-phenylenediamide-urea reagent (OPD tablet; Sigma Co., MO; dissolved in 15 mL of distilled water) for 2 min. The reaction was terminated by adding 75 µL of 0.5 N sulfuric acid and the absorbance was measured at 490 nm. In addition, to determine antibody titers, gangliosides GM1 and GQ1bα, 25 ng each, were placed in individual wells of an ELISA plate and the test serum was added by diluting 1∶400 to 1∶12,800 with 1% BSA/PBS and then examined as described above. Anti-ganglioside antibody was represented as the highest serum dilution (1 x as specified) at which the background of OD_490 nm_ subtracted was 0.25 (for IgM) or 0.15 (for IgG), respectively.

### High-performance Thin-layer Chromatography (HPTLC)-immunostaining

HPTLC-immunostaining was performed as previously described [15]. Briefly, one microgram of a ganglioside (GM1, GD1a, GD1b, GT1b, or GQ1bα) was applied onto an HPTLC plate. In a separate experiment, one microgram of a GSL (GM1, GD3, and GQ1b, or SGPG) was applied onto an HPTLC plate. The plate was developed with chloroform: methanol: 0.2% aqueous CaCl_2_•2H_2_O (55∶45:10, v/v), left to air-dry, and was then immersed in 0.05% polyisobutyl methacrylate in n-hexane for 1 min. After drying, the plate was incubated for 30 min with 0.3% gelatin (Sigma, MO) in 0.5 M PBS (pH 7.4). After washing with PBS, the plate was incubated with sera of demented patients and normal controls for 2 hr at room temperature. After washing five times with PBS, the plate was incubated with peroxidase-conjugated anti-human IgM (1∶2,000, diluted with gelatin-PBS) for 2 hrs. After further washing with PBS, bands were visualized with 3,3′-diaminobenzidine tetrahydrochloride (Sigma, MO), 0.1% imidazole and 0.01% hydrogen peroxide [16].

### Statistical Analysis

Anti-GSL antibody activities in each serum sample were determined at least five times by ELISA. The combined results are expressed as mean ± SD. The data are analyzed by one-way analysis of variance (ANOVA) followed by post-hoc analysis for multiple comparison (Tukey’s test). Statistical significance is established at *p*<0.05.

## Results

In the present study, sera from 27 individuals (10 with AD, 6 with VD, and 11 age-matched controls without dementia) were subjected to anti-GSL antibody determination. Diagnosis of AD and VD was based on criteria of the DSM-IV and MMSE. Table 1 shows that patients were matched by age (years ± SD): AD (78.2±5.9), VD (79.5±5.6), control (72.5±11.0). These results showed no significant differences. MMSE is a widely used test of cognitive functions including orientation, attention, memory, language, and visual-spatial skills. As shown in Table 1, when compared to age-matched controls (score ± SD; 27.7±1.4), the MMSE revealed low scores, between 5 and 20 (13.3±4.9) in AD patients and between 13 and 23 (16.6±3.9) in VD patients, respectively (*p*<0.05). Table 2 shows the absorption of anti-GSL antibodies (OD_490mμ_) and percentages of positive cases with demented patients and age-matched controls using sera diluted at 1∶400. Although the control sera had natural autoantibodies to GSLs, especially to ganglio-N-tetraosyl ceramides [17], all sera of the demented patients were found to have higher titers for anti-GSL antibodies compared to the age-matched controls, including anti-GalCer, -GM1, -GT1b, -GQ1b as well as -SGPG. In VD, anti-GM1, -GT1b, -GQ1b, and -GQ1bα antibodies of the IgM type were elevated. Table 2 also shows the percentage of positive cases of elevated IgM antibody titers, e.g., in AD, anti-SGPG (100%), -GM1(100%), -GD1b(100%), -GT1b(100%), and -Q1bα (90%, 9 of 10 cases) antibodies, and in VD, anti- GA1 and -GM1 (83%, 5 of 6 cases), -GT1b (100%), -GQ1b (67%, 4 of 6 cases), and -GQ1bα (100%) antibodies. However, anti-SGPG and -GD1b antibodies (IgM) seemed to be non-specific, since 73% of aged-matched controls also had these antibodies. On the other hand, anti-GT1b antibodies (IgG) were elevated in AD (90% or 9 of 10 cases) and VD (100%). Anti-GQ1bα antibodies (IgG) were elevated in AD (40%, 4 of 10 cases) and VD (67%, 4 of 6 cases).

**Table 1:** Characteristic of all cases.

	Case	Age	Sex	MMSE
**AD**	1	73	M	5
	2	87	F	10
	3	79	F	10
	4	86	F	18
	5	81	F	11
	6	81	M	19
	7	61	M	15
	8	80	F	6
	9	71	F	20
	10	83	M	19
	mean ± SD	78.2±7.9		13.3±5.6*
**VD**	11	82	F	13
	12	68	M	13
	13	75	F	13
	14	91	F	22
	15	82	F	16
	16	79	M	23
	mean ± SD	79.5±7.7		16.7±4.7*
**C**	17	81	M	25
	18	68	M	27
	19	84	M	26
	20	68	M	28
	21	52	M	30
	22	92	M	26
	23	68	M	28
	24	78	F	28
	25	76	M	27
	26	64	M	30
	27	67	M	30
	mean ± SD	72.5±11.0		27.7±1.7

AD, Alzheimer's disease; VD, vascular dementia; C, controls without dementia.

MMSE, mini-mental state test;

* ,p<0.05 vs C.

**Table 2:** Absorption and percentages of positive cases of anti-glycosphingolipid antibodies.

IgM		anti-GD3	anti-GA1	anti-GM1	anti-GD1a	anti-GD1b	anti-GT1b	anti-GQ1bα
**AD (n = 10)**	Absorbtion (OD490 nm)	0.42±0.20	0.42±0.18	0.44±0.15*	0.40±0.21	0.44±0.17	0.47±0.17*	0.43±0.12*
	Percentage of positive cases	90.0	80.0	100.0	80.0	100.0	100.0	90.0
**VD (n = 6)**	Absorbtion (OD490 nm)	0.34±0.25	0.45±0.29	0.49±0.24*	0.27±0.31	0.32±0.36	0.57±0.22*	0.49±0.20*
	Percentage of positive cases	50.0	83.3	83.3	33.3	33.3	100.0	100.0
**C (n = 11)**	Absorbtion (OD490 nm)	0.26±0.11	0.30±0.17	0.26±0.12	0.23±0.10	0.33±0.13	0.27±0.09	0.23±0.09
	Percentage of positive cases	45.5	45.5	36.4	54.5	72.7	45.5	27.3
**IgM**		**anti-SGPG**	**anti-GM3**	**anti-GQ1b**	**anti-GalCer**	**anti-LacCer**	**anti-Sul**	
**AD (n = 10)**	Absorbtion (OD490 nm)	0.53±0.17	0.46±0.27	0.24±0.16*	0.37±0.20	0.22±0.17	0.39±0.19	
	Percentage of positive cases	100.0	90.0	50.0	70.0	40.0	80.0	
**VD (n = 6)**	Absorbtion (OD490 nm)	0.40±0.25	0.33±0.26	0.30±0.19*	0.32±0.16	0.14±0.14	0.31±0.16	
	Percentage of positive cases	66.7	50.0	66.7	66.7	33.3	50.0	
**C (n = 11)**	Absorbtion (OD490 nm)	0.36±0.14	0.30±0.22	0.06±0.08	0.27±0.24	0.19±0.21	0.32±0.21	
	Percentage of positive cases	72.7	54.5	0.0	36.4	27.3	54.5	
**IgG**		**anti-GD3**	**anti-GA1**	**anti-GM1**	**anti-GD1a**	**anti-GD1b**	**anti-GT1b**	**anti-GQ1bα**
**AD (n = 10)**	Absorbtion (OD490 nm)	0.14±0.05	0.12±0.08	0.15±0.10	0.15±0.08	0.15±0.07	0.19±0.04	0.15±0.06*
	Percentage of positive cases	40.0	30.0	40.0	40.0	40.0	90.0	40.0
**VD (n = 6)**	Absorbtion (OD490 nm)	0.16±0.10	0.11±0.08	0.14±0.07	0.15±0.09	0.15±0.08	0.24±0.05*	0.14±0.06
	Percentage of positive cases	33.3	33.3	50.0	50.0	50.0	100.0	66.7
**C (n = 11)**	Absorbtion (OD490 nm)	0.09±0.04	0.12±0.08	0.10±0.07	0.08±0.04	0.11±0.08	0.14±0.08	0.07±0.04
	Percentage of positive cases	9.1	18.2	18.2	0.0	18.2	36.4	0.0
**IgG**		**anti-SGPG**	**anti-GM3**	**anti-GQ1b**	**anti-GalCer**	**anti-LacCer**	**anti-Sul**	
**AD (n = 10)**	Absorbtion (OD490 nm)	0.18±0.05	0.16±0.10	0.12±0.06*	0.09±0.03	0.04±0.02	0.12±0.08	
	Percentage of positive cases	70.0	60.0	30.0	0.0	0.0	30.0	
**VD (n = 6)**	Absorbtion (OD490 nm)	0.17±0.10	0.13±0.08	0.10±0.06	0.05±0.03	0.02±0.02	0.13±0.09	
	Percentage of positive cases	50.0	33.3	16.7	0.0	0.0	50.0	
**C (n = 11)**	Absorbtion (OD490 nm)	0.12±0.07	0.11±0.05	0.04±0.04	0.06±0.04	0.06±0.07	0.07±0.04	
	Percentage of positive cases	36.4	18.2	9.1	0.0	9.1	0.0	

Values were represented the absorption (OD490 nm) ± SD. Anti-GSL antibody activities in each serum. Percentage of positive cases was cut off the absorption lower than 0.25 for IgM and 0.15 for IgG. AD, Alzheimer's disease (n = 10); VD, vascular dementia (n = 6); C, controls without dementia (n = 11),

* ,p<0.05.

The statistical data revealed that the sera of demented patients had significantly higher titers of the IgM type of anti-GSL antibodies, especially activities against GM1, GT1b, GQ1b, and GQ1bα than the age-matched controls (*p*<0.05). In AD patients, anti-GT1b, -GQ1b, and -GQ1bα, antibodies (IgG type) were elevated. Interestingly, anti-GT1b antibodies were also significantly elevated in VD.

Table 3 shows a summary of the titers of anti-GM1 and -GQ1bα antibodies in the sera of demented patients and aged-matched controls. Demented patient’s sera had between 400 and 3,200 times higher titers of anti-GM1 and -GQ1bα antibodies (IgG type) than those in the corresponding controls, although most of the control sera had between 400 and 800 times lower titers, three out of 11 aged-matched controls had high titers of anti-GQ1bα (IgM type; 1,600x). Two out of ten AD patients had significantly higher titers of anti-GM1 and -GQ1bα antibodies (IgM type; >12,800). In addition, two patients with VD had higher titers of anti-GM1 and -GQ1bα antibodies (IgM type; 6,400x) than the corresponding controls.

**Table 3:** Titers of anti-glycosphingolipid, GM1 and GQ1bα antibodies in sera of demented patients, and age-matched controls.

Case		IgG		IgM	
		GM1	GQ1bα	GM1	GQ1bα
**AD**	1	400	1,600	800	1,600
	2	400	800	800	1,600
	3	800	800	1,600	1,600
	4	6,400	6,400	6,400	3,200
	5	1,600	3,200	3,200	6,400
	6	800	800	1,600	1,600
	7	800	800	1,600	1,600
	8	400	800	1,600	3,200
	9	3,200	3,200	>12,800	>12,800
	10	3,200	3,200	>12,800	>12,800
**VD**	11	1,600	3,200	6,400	6,400
	12	3,200	1,600	3,200	6,400
	13	1,600	3,200	1,600	1,600
	14	3,200	3,200	3,200	6,400
	15	1,600	1,600	1,600	1,600
	16	1,600	1,600	1,600	3,200
**C**	17	<400	400	400	400
	18	<400	<400	400	<400
	19	<400	<400	800	800
	20	1,600	800	1,600	800
	21	<400	400	400	400
	22	800	800	800	400
	23	800	400	400	400
	24	400	400	800	800
	25	<400	<400	400	400
	26	<400	<400	400	1,600
	27	<400	<400	800	800

AD, Alzheimer's disease; VD, vascular dementia; C, controls without dementia.

**Figure 2:**
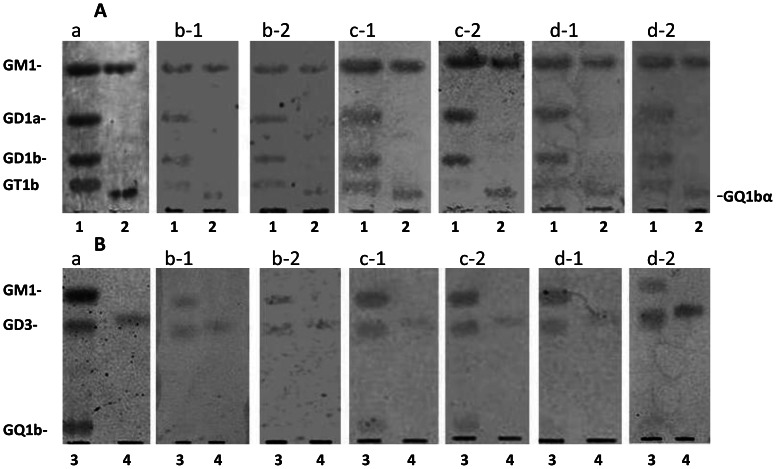
HPTLC-immunostaining of brain gangliosides, such as GM1, GD1a, GD1b, GT1b, and GQ1bα (plate A) and GM1, GD3, GQ1b, and SGPG (plate B). Lane 1, 1 µg each of GM1, GD1a, GD1b, and GT1b; 2, 1 µg of GM1 and GQ1bα; 3, 1 µg each of GM1, GD3, and GQ1b; and 4, 1 µg of SGPG. a, orcinol sulfuric acid staining; b, c, and d, immunostaining; b-1 and b-2, control sera; c-1 and c-2, AD sera (Alzheimer’s disease); d-1 and d-2, VD sera (vascular dementia). Plates A and B were developed with CH_3_Cl_3_:CH_3_OH:0.2%CaC_2_•2H_2_O (55∶45:10, v/v).

Fig. 2 shows typical HPTLC-immunostaining of anti-brain ganglioside (ganglio-N-tetraosyl ceramides) antibodies (IgM type) (plate A) and anti-GM1, -GD3, -GQ1b, and –SGPG antibodies (IgM type) (plate B). All aged-matched control sera had weak natural serum autoantibodies against brain gangliosides (plate A). However, HPTLC-immunostaining revealed that anti-ganglioside antibodies not specific for GM1 ganglioside were significantly elevated in the sera of demented patients. In general, the sera of demented patients had higher reactivity for not only GM1 ganglioside, but also other gangliosides (GD1a, GD1b, GT1b and GQ1b) with varying reactivities. Interestingly, some sera of demented patients had extremely higher reactivity to a cholinergic antigen, GQ1bα, compared with controls. Although control sera had weak activities to GM1, GD3, and SGPG (plate B), the sera of demented patients had higher levels of activity against GM1, GD3 and SGPG with variable reactivity.

## Discussion

It has been hypothesized that serum antibodies are involved in the pathogenesis of AD and certain related diseases. For example, Mosek et al. [18] showed that, based on ELISA analysis, anti-phospholipid (cardiolipin) antibodies in the blood were elevated in 5 of 87 (6%) of demented patients compared with 69 controls. All the patients with high anti-phospholipid antibody levels were diagnosed clinically as dementia with AD. In particular, autoantibodies against organ-specific central nervous system (CNS) antigens were increased in the patients compared with controls [8], [19]. Serum antibodies against a series of antigens, including an organ-specific CNS antigen and the neurotransmitter serotonin, were investigated in 22 patients with AD (n = 15) and other age-related dementias (n  = 7) by indirect immunofluorescence assay and ELISA. Patients with dementia showed an increase of antibody-positive sera against nuclear antigen, gastric parietal cells, CNS antigen, gangliosideGM1, laminin, and keratin. Interestingly, only AD patients exhibited antibodies against CNS antigens. However, these results were not sufficiently specific nor sensitive enough to be useful as a reliable diagnostic indicator [19]. With respect to anti-ganglioside antibodies, Chapman et al. [8] first reported a significant level of antibodies specific to GM1 in patients with AD when compared to normal age-matched controls. A high titer of antibodies against GM1 was also found in patients with multi-infract dementia and PD with dementia, but not in non-demented patients with other neurodegenerative diseases. In addition, Hatzifilippou et al. [20] recently reported a correlation between high titers of serum anti-GM1 antibody (IgM type) and the most severely demented AD patients. Eighty-two percent of demented patients with AD revealed increased titers of anti-GM1, but only 18.2% of patients with polyneuropathies did. Fifty-nine percent of the patients suffering from VD also showed increased titers of anti-GM1. The findings of this study are indicative of a possible correlation between the levels of anti-GM1 and the severity of dementia, especially the vascular type. These studies suggest that evaluation of the titer of anti-GM1 antibody in demented patients may provide a useful correlate with the severity of dementia. In the present study, we found that demented patients had specific antibody reactivity not only against GM1, but also against GD1a, GD1b, GT1b, and GQ1b. The anti-GM1 antibody activities are consistent with the loss of neuronal functions.

With respect to GSLs, it should be noted that normal sera might have natural antibodies against ganglio-N-tetraosyl gangliosides (GM1, GD1b, etc) as well as asialo-GM1 (GA1) [17], [21], [22], [23]. Ganglioside-specific IgM antibodies present in the sera from healthy volunteers are thought to represent naturally occurring antibodies recognizing microbial GSLs [21] and appeared to be cross-reacting antibodies originally directed to a GA1-like structure recognizing the Galβ1-3GalNAc epitope [24]. In the present study, we used the HPTLC-immunostaining method and found that when sera were tested at high concentrations, e.g., only diluted to 1∶50 to 1∶100, they recognized all ganglio-N-tetraosyl gangliosides and no differences could be found between demented patients and normal subjects (data not shown). On the other hand, when sera were diluted 1∶500, the activities against ganglio-N-tetraosyl gangliosides (IgM type; GM1, GD1a, GD1b, GT1b, GQ1bα) were greatly diminished, and only at or above this dilution could significant differences in antibody titer be detected between demented patients and age-matched normal subjects. Sera of demented patients had particularly higher immunoreactivity against brain gangliosides than those in age-matched controls (Table 2). Our results are therefore at variance with those of Chapman et al. [8] who reported high titers of antibodies only against GM1, but not other gangliosides (GD1a, GD1b, GT1b, and GQ1b). Interestingly, in some demented sera, the IgM type of anti-GQ1bα antibodies was notably elevated as well as anti-GT1b and anti-GQ1b antibodies. These autoantibody titers had only weak reactivity in age-matched controls. The titers of IgG type GSL antibodies were significantly lower than those of the IgM type, but it was noted that serum anti-GT1b antibodies were elevated in patients with dementia.

The blood-brain barrier (BBB) is known to be formed by the brain capillary endothelium. The major function of the BBB is to exclude large-molecules, such as antibodies and blood cell components from entering into the brain. However, the BBB becomes more permeable as the result of inflammation, which causes certain macromolecules to penetrate into the brain. The BBB breakdown and increased permeability are critical events in the pathogenesis of many neurological diseases including multiple sclerosis (MS) [25], AD [26], and Parkinson's disease (PD) [27] and may be involved in disease progression [27], [28]. In MS, neurodegeneration develops in association with inflammation, and demyelination [29] and axonal degeneration, which are the major determinants of progressive neurological disability [30]. Several studies have demonstrated that sera from patient with MS had elevated titers of anti-GSL antibodies, such as anti- GM1 [31], [32], anti-asialo-GM1 [31], anti-GD1a [31], [33], anti-GM3 [34], [35], anti-GD2 [36], anti-GM4 [22], anti-GalCer [22], [37], anti-GQ1b [35], and anti-sulfatide [38]. Several studies have demonstrated a positive correlation between axonal damage and the degree of inflammation in active cerebral MS lesions. In addition, sera of some patients with PD had high titer anti-GM1 antibodies [39]. AD is also known to be associated with degenerative changes to blood vessels that may compromise the integrity of the BBB [40]. Thus, inflammatory events may be implicated in the breakdown of the vasculature in AD. In the present study, the sera from demented patients had several high-titer anti-ganglioside antibodies that were not specific antibodies, which seemed to cause leakage of the BBB resulting in neuropathological damage [41]. Although it is unclear whether anti-ganglioside antibodies can cause or result from axonal damage they may certainly serve as a marker of this process [34], [41]. Experimental studies have reported that anti-ganglioside antibodies can increase the permeability of the BBB in a concentration-dependent and complement-independent manner, resulting in disruption of BBB [42] and the subsequent induction of neuromuscular block by binding to neuronal gangliosides in the neuromuscular junction [43], [44], or inhibition of axonal regeneration by engaging cell surface gangliosides [7].

GQ1bα is one of the so-called Chol-1 antigens that are specific markers of cholinergic neurons and normally are minor species in the brain [9], [45]. In rat brain, Chol-1α antigens are first detected between the 10^th^ and 20^th^ day of postnatal age, reaching adult levels at 50 days of normal development [45]. However, the expression of Chol-1 antigens in rat brain regions, such as the hippocampus, is known to be developmentally regulated, and their concentrations increase with aging [45]. In mouse brain, their expression is present only during early prenatal stages characterized by rapid neuronal differentiation, presumably during development of the cholinergic neuronal system [46]. In our recent study, we found the expression level of GQ1bα was significantly elevated in brains of a transgenic (Tg) mouse model of AD that co-expresses mouse/human chimeric amyloid precursor protein (APP) with Swedish mutation (K595N/M596L) (APPswe) as compared with those in wild-type mouse brains [47]. There is a distinct possibility that up-regulation of the expression of GQ1bα ganglioside in AD mouse brain could be a compensatory mechanism for the age-related decline of cholinergic function underlying AD. These findings suggest that abnormalities in gangliosides in the brains in individuals with AD may reflect developmental disturbances.

These observations may reflect a specific change in ganglioside metabolism. This possibility is in line with the report from Chapman et al. [48] who suggested that AD is associated with degenerative changes in the basal nuclei of the forebrain that provide most of the cholinergic input to the cortex and hippocampus and with a reduction in presynaptic cholinergic parameters in these areas. In fact, Chapman et al. [48] reported the presence of antibodies in sera of AD patients that bind specifically to cholinergic neurons. Foley et al. [49] also reported the correlation of the presence of antibodies to cholinergic neurons and the pathogenesis of AD.

In conclusion, our results show a particular change in ganglioside metabolism is associated with the neurodegenerative processes underlying AD and other causes of dementia. The presence of serum anti-GSL antibody activities in demented patients is consistent with the loss of neuronal function. Since Chol-1α gangliosides are located in cholinergic nuclei neurons in the gray matter, septal nucleus, and inter-peduncular nucleus of the mammalian brain, an elevation of anti-GQ1bα in demented patient’s sera may contribute to the modulation of cholinergic neurotransmission, resulting in brain cognitive dysfunction such as loss of memory and inability for learning in AD and related dementia. Anti-ganglioside antibodies may reflect specific changes associated with neuronal lesions in AD and related dementia.
